# Cerebrospinal Fluid PKR Level Predicts Cognitive Decline in Alzheimer’s Disease

**DOI:** 10.1371/journal.pone.0053587

**Published:** 2013-01-08

**Authors:** Julien Dumurgier, Francois Mouton-Liger, Pauline Lapalus, Magali Prevot, Jean-Louis Laplanche, Jacques Hugon, Claire Paquet, for the Groupe d’Investigation du Liquide Cephalorachidien (GIL) Study Network

**Affiliations:** 1 Clinical and Research Memory Center, Paris Nord Ile de France Saint Louis Lariboisière Fernand Hospital, AP-HP, University of Paris Diderot, Paris, France; 2 Institut du Fer à Moulin, Inserm UMR-S 839, Paris, France; 3 Department of Biochemistry Saint Louis Lariboisière Fernand Hospital, AP-HP, University of Paris Diderot, Paris, France; Banner Alzheimer's Institute, United States of America

## Abstract

The cerebrospinal fluid (CSF) levels of the proapoptotic kinase R (PKR) and its phosphorylated PKR (pPKR) are increased in Alzheimer’s disease (AD), but whether CSF PKR concentrations are associated with cognitive decline in AD patients remain unknown. In this study, 41 consecutive patients with AD and 11 patients with amnestic mild cognitive impairment (aMCI) from our Memory Clinic were included. A lumbar puncture was performed during the following month of the clinical diagnosis and Mini-Mental State Examination (MMSE) evaluations were repeated every 6 months during a mean follow-up of 2 years. In AD patients, linear mixed models adjusted for age and sex were used to assess the cross-sectional and longitudinal associations between MMSE scores and baseline CSF levels of Aβ peptide (Aβ 1-42), Tau, phosphorylated Tau (p-Tau 181), PKR and pPKR. The mean (SD) MMSE at baseline was 20.5 (6.1) and MMSE scores declined over the follow-up (-0.12 point/month, standard error [SE] = 0.03). A lower MMSE at baseline was associated with lower levels of CSF Aβ 1–42 and p-Tau 181/Tau ratio. pPKR level was associated with longitudinal MMSE changes over the follow-up, higher pPKR levels being related with an exacerbated cognitive deterioration. Other CSF biomarkers were not associated with MMSE changes over time. In aMCI patients, mean CSF biomarker levels were not different in patients who converted to AD from those who did not convert.These results suggest that at the time of AD diagnosis, a higher level of CSF pPKR can predict a faster rate of cognitive decline.

## Introduction

Alzheimer’s disease (AD) is classically marked by the progressive occurrence of memory disturbances followed by aphasia, apraxia and agnosia associated with behavioral symptoms [1]. It is difficult to predict clinically the rate of cognitive decline in affected patients [2]. The brain lesions in AD are characterized by senile plaques made of extracellular accumulated Aβ peptides, neurofibrillary tangles formed by hyperphorylated tau protein and synaptic and neuronal losses [3]. Over the past several years, the analysis of cerebrospinal fluid (CSF) biomarkers such as Aβ 1-42, Tau and phosphorylated Tau (p-Tau 181) has improved the accuracy of the clinical diagnosis, even at the early phase of the disease [4]. These CSF biomarkers reflect the magnitude of neuropathological lesions detected in AD brains [5], [6]. Several cofounding factors, such as vascular lesions [7] or the cognitive reserve [8] can influence the evolution of cognitive signs in AD and may delay or precipitate the early symptoms. So far, it has been very difficult to find out a reliable biological marker in the blood or in the CSF that could predict the slope of cognitive deterioration in affected patients. The double-stranded RNA dependent protein kinase (PKR) is a ubiquitous cellular kinase that controls protein synthesis by phosphorylating the eukaryotic initiation factor 2α. PKR also controls viral infection, inflammation and when activated by auto-phosphorylation is a major factor of cell death [9]. Activated PKR is increased in AD brains [10] and PKR activation via Aβ 1-42, can also lead to the phosphorylation of Tau protein and during oxidative stress can modify β –secretase 1 (BACE1) protein levels, one of the main enzyme implicated in the formation of Aβ peptides [10]–[12]. We have recently shown that the levels of phosphorylated PKR (pPKR) were increased in the CSF of patients with AD and amnestic mild cognitive impairment (aMCI) compared to neurological disease controls, and that pPKR levels correlate with p-Tau 181 levels in AD patients [13]. All these results can argue in favor of a possible role of PKR in AD pathophysiology.

The goal of the present study was to determine in a longitudinal cohort of AD and aMCI patients the possible links between the rate of cognitive decline and the initial levels of CSF biomarkers including PKR and pPKR. Our results show that CSF pPKR concentration can predict the future cognitive decline in AD patients.

## Materials and Methods

### Patients

41 consecutive patients with a diagnosis of AD have been recruited from our outpatient Memory Clinic between January 2010 and January 2011, as previously described [13]. AD diagnosis was made according to NINCDS-ADRDA criteria [14] and was performed by a team of neurologists and neuropsychologists specialized in cognitive disorders. All patients were treated by cholinesterase inhibitors and/or by memantin when appropriate. Every 6 months, patients underwent neurological exams and neuropsychological assessments including a Mini-Mental State Examination (MMSE) evaluation. In addition, we also included 11 aMCI patients from our initial discovery cohort and we established the number of MCI patients who converted to AD at the end of the follow-up survey (June 2012), according to the NINCDS-ADRDA criteria [14].

This work has been approved by the Ethics Committee of Paris University Hospital (Bichat Hospital) and all patients or caregivers gave their written informed consent for this study. Next of kin, care takers or guardians consented on the behalf of participants whose capacity to consent was compromised. Usually, patients with mild AD forms signed the consent, for moderate AD forms patients and care givers signed the consent and in severe AD forms care takers signed the consent.

### CSF Procedures

Lumbar punctures were performed in fasting patients in the following month after the clinical diagnosis. CSF was collected in 12 mL polypropylene tubes. Within 2 hours, CSF samples were centrifuged at 1800 g for 10 minutes at 4°C. An appropriate part of CSF was used for routine analysis, including total cell count, bacteriologic exam, and total protein and glucose levels. CSF was aliquoted in polypropylene tubes of 500 µL and stored at −80°C until further analysis. CSF Aβ 1-42, Tau, and p-Tau 181 were measured with Innotest sandwich enzyme-linked immunosorbent assay (ELISA) according to manufacturer’s procedures (Innogenetics, Ghent, Belgium). Positivity criteria for biomarkers were defined by anomalies of Aß and T-tau or p181tau levels, according to the cutoffs of our center (Aß<500 pg/mL; T-tau>300 pg/mL; p181tau>65 pg/mL). PKR and pPKR CSF levels were analyzed by western blots procedures as previously reported (13). Results of PKR and pPKR are expressed in optical density units (ODU). All biological analyses were done in a single hospital laboratory. The biological team involved in the CSF analysis was unaware of the clinical diagnosis. The quality of CSF evaluations was validated by a European Consortium (Alzheimer’s Association Quality Control [QC] Program for CSF Biomarkers).

### Statistical Analysis

Linear mixed models were used to study the relationship between baseline levels of CSF biomarkers and repeated measurements of MMSE scores. The intercept and the slope (time) of the models were treated as random effects, allowing them to vary between individuals, with an unstructured covariance matrix. Time in months since baseline was included as a continuous linear term after verification that a quadratic term did not provide a better fit. We used z-scores to estimate standardized regression coefficients β that allow comparing the strength of the relations between MMSE and the different biomarkers. Models included one of the CSF biomarkers, age, sex, time and the interaction between time and the different parameters.Results were also shown using tertiles of patients according to CSF pPKR at baseline. In a complementary analysis, 11 patients with amnestic MCI, coming from our previously reported cohort [13], were assessed for conversion. These patients were categorized into two groups; those who have converted to AD (converters) or those who have not converted (non-converters) at the end of the follow-up period. Mean levels of the various CSF biomarkers between these 2 groups were compared using Kruskal-Wallis non parametric analysis.

P-values were two-tailed and values ≤0.05 considered as statistically significant. Analyses were performed using SAS 9.2 (SAS Institute, Cary, North Carolina, USA).

## Results

Baseline characteristics of the study cohort are depicted in Table 1. Forty one AD patients were followed during a mean (SD) period of 25.7 (4.5) months. During this period, the mean (SD) number of MMSE evaluations was 4.5 (2.9) per patient. Their mean (SD) age was 69.9 (7.4) years, 61% of them were women, and their mean (SD) MMSE score was 20.5 (6.1).

**Table 1:** Baseline characteristics of the study sample.

Characteristics	AD patients, n = 41
Age, years, mean (SD)	69.9 (7.4)
Women, n (%)	25 (61)
MMSE, mean (SD)	20.5 (6.1)
CSF biomarkers, pg/mL, mean (SD)	
Aβ 1-42	423.4 (152.2)
Tau	597.7 (340.2)
p-Tau 181	108.1 (54.3)
p-Tau 181/Tau ratio	0.20 (0.04)
CSF PKR, ODU, mean (SD)	
T-PKR	62.8 (19.3)
pPKR	88.5 (36.0)
Follow-up, months, mean (SD)	25.7 (4.5)
Number of MMSE evaluations, mean (SD)	4.5 (2.9)

Table 2 shows the results of age and sex adjusted linear mixed model estimates concerning the relationships between baseline CSF biomarkers and MMSE scores at baseline and during the follow-up survey. Lower levels of CSF Aβ 1-42 and p-Tau 181/Tau ratio were associated with lower baseline MMSE score, with standardized estimates β (SE) respectively equal to 2.5 (0.87) (p = 0.007) and 2.0 (0.97) (p = 0.048). In the longitudinal analysis, the mean (SE) decline of MMSE was −0.12 (0.03) point per month (p<0.001). Higher baseline level of CSF pPKR was associated with a more marked decline of MMSE over follow-up (β[SE] = −0.065 (0.032), p = 0.043). Other CSF biomarkers were not associated with MMSE change.

**Table 2:** General linear mixed models estimates of the relationship between baseline CSF biomarkers and MMSE score at baseline and over follow-up.

CSF biomarkers	Estimate (SE)^a^	*P* a
**MMSE score at baseline**		
Intercept	20.9 (1.1)	<0.001
Aβ 1-42	2.5 (0.87)	0.007
Tau	0.067 (0.96)	0.95
p-Tau 181	0.55 (0.81)	0.50
p-Tau 181/Tau ratio	2.0 (0.97)	0.048
T-PKR	−0.73 (0.96)	0.45
pPKR	−0.83 (0.89)	0.35
**Change in MMSE over the follow-up**		
Time (in month)	−0.122 (0.032)	<0.001
Aβ 1-42×Time	0.002 (0.033)	0.94
Tau×Time	−0.037 (0.037)	0.32
p-Tau 181×Time	−0.042 (0.041)	0.30
p-Tau 181/Tau ratio	0.007 (0.035)	0.84
T-PKR×Time	−0.009 (0.037)	0.81
pPKR×Time	−0.065 (0.032)	0.043

a Standardized estimates with their standard error (SE) computerized from mixed models adjusted for age, sex, CSF biomarker, and their interactions with time (one model per biomarker).

**Figure 1:**
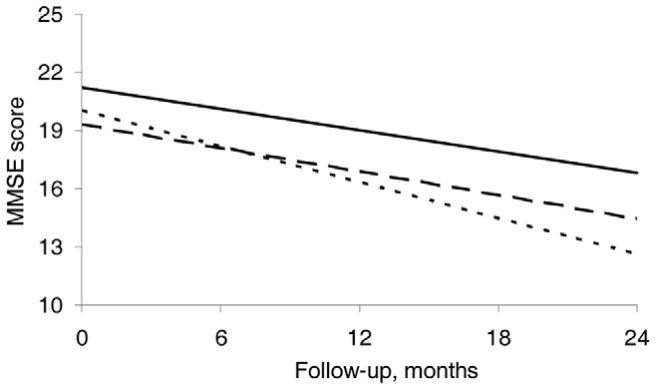
Predicted trajectories of MMSE score according to tertiles of CSF phosphorylated PKR. Mixed model is adjusted for age, sex, tertiles of CSF pPKR and their interactions with time in months. Solid line: lower tertile of pPKR (0–65 ODU), long-dashed sline: middle tertile of pPKR (65–100 ODU), short-dashed line : upper tertile of pPKR (>100 ODU). Cognitive decline is more pronounced in patients with high levels of CSF pPKR (short-dashed line).

The Figure 1 illustrates the evolution of MMSE scores according to tertiles of CSF pPKR at baseline. Patients in the higher tertile (pPKR>100 optical density units) tended to have an exacerbated decline of MMSE compared to those in the lower tertile (pPKR<65 ODU) (p = 0.09).

Table 3 presents the results of mean biomarker levels in the 2 groups of aMCI patients. Their mean (SD) age was 76.9 (10.5) years, 7 were women, their mean (SD) MMSE was 24.1. Seven of them converted to AD, 4 were non-converters. None of these CSF biomarker reached statistical significance, however, CSF total PKR was the most discrimant biomarker between the 2 groups (converters, mean [SD] T-PKR = 94.8[37.6], non converters, mean [SD] T-PKR = 67.7[44.7], p = 0.09).

**Table 3:** CSF biomarker levels in converters and non-converters MCI patients.

	Patients with MCI				
	All	Converters	Non converters		
CSF biomarkers	(N = 11)	(N = 7)	(N = 4)	Statistic^a^	P-value^a^
Aβ 1-42, pg/mL, mean (SD)	608.7 (276.4)	657.7 (281.6)	523.0 (283.9)	1.76	0.18
Tau, pg/mL, mean (SD)	316.1 (124.2)	329.6 (45.0)	292.5 (214.9)	1.75	0.19
p-Tau 181 pg/mL mean (SD)	63.1 (18.8)	67.2 (14.3)	56.0 (25.8)	0.57	0.45
p-Tau 181/Tau ratio, mean (SD)	1.10 (0.37)	1.16 (0.36)	1.00 (0.43)	0.89	0.34
T-PKR, ODU, mean (SD)	75.3 (21.6)	82.2 (20.3)	63.3 (20.6)	2.89	0.09
pPKR, ODU, mean (SD)	84.9 (40.4)	94.8 (37.6)	67.7 (44.7)	1.75	0.19

a Kruskal-Wallis Test.

## Discussion

Our results show that the level of CSF pPKR is associated with a more pronounced cognitive decline, as measured with the MMSE, in a cohort of patients newly diagnosed with AD. While CSF Aβ 1-42 levels and p-Tau 181/Tau ratio were cross-sectionally associated with MMSE score at the time of the diagnosis, pPKR was the only biomarker to be linked to the cognitive decline over the follow-up survey.

Several previous studies have reported an association between CSF biomarkers and cognitive change in AD, and results remain debated. In a previous study, one cluster of AD patients with extreme levels of CSF biomarkers were characterized by a faster progression of their cognitive deficit, no response to cholinesterase inhibitor treatment, and a higher mortality [15]. Another study using quintiles of patients found that CSF p-Tau 181/Tau ratio and Aβ 1-42 levels were associated with cognitive decline assessed by repeated MMSE, not with baseline MMSE [16]. CSF biomarkers have also been associated with longitudinal brain atrophy assessed by volumetric MRI [17]–[19]. Other potential CSF biomarkers of neuronal damage, such as Visinin-like protein-1, have also been linked to the rate of cognitive decline using terciles of patients [20], [21].

In a previous study we have shown that CSF PKR and pPKR levels were increased in AD patients as compared to neurological disease controls [13]. The first question that can be addressed is why pPKR can predict the global cognitive decline in AD patients. As mentioned earlier, PKR, once activated, is a pro-apoptotic kinase that accumulates in degenerating neurons [22] and patients with very high CSF levels of pPKR could simply be affected by a more widespread neuronal death. Another factor that could contribute to exacerbated degradation of cognition is neuroinflammation. High levels of inflammatory cytokines with microglial activation have been detected in AD brains [23] and PKR is known to play a role in the production of pro-inflammatory cytokines through the induction of the transcription factor NKκB. [24] It is possible to assume that PKR, which is activated by Aβ [22], could contribute to the chronic over production of cytokines by immune competent cells such as microglia leading to detrimental consequences in neurons. A final factor that could explain our results is the relation between Tau phosphorylation and activated PKR [11]. The phosphorylation of PKR can indirectly lead to the phosphorylation of Tau protein and in turn could accentuate the toxic consequences of neurofibrillary tangles in affected neurons.

The second question that can be posed is to know if analyzing CSF pPKR levels could be useful at the onset of the clinical disease. Although classical biomarkers are very useful to predict the clinical outcome in patients with MCI [4], it appears that in demented patients, their usefulness to predict the cognitive decline in longitudinal surveys, is not well established. In addition, to dispose of a possible surrogate biomarker in clinical trials, might be a way to objectively assess the efficacy of a pharmacological approach. Further studies in other cohorts of patients will be necessary to confirm our findings and to establish the exact usefulness of the evaluation of CSF pPKR. Similarly, the assessment of CSF Aβ 1-42 and pPKR in preclinical AD might help to understand the relations between these two biomarkers with imaging studies [25].

There are some limitations in our study. Firstly, the number of patients included in our study remains limited. The lack of association between CSF biomarkers levels and conversion from MCI to AD we observed may be probably linked to the small number of MCI patients in our sample. A larger multicentric study including other types of dementia as well as MCI patients is needed to confirm the link between pPKR and the cognitive decline. Secondly, other indicators such as volumetric MRI or PET scan DFG glucose could also be compared with pPKR in future studies. Thirdly, the patients have been followed up during a mean period of time of 26 months. This is obviously limited and longer surveys are needed in order to detect exacerbated cognitive decline. We did not include the neurological controls of our initial discovery cohort of patients because those patients were suffering from an heterogenous group of diseases with various clinical evolutions. In addition, apolipoprotein E genotyping should be performed in future studies to determine a possible influence of ApoE4 genotype on pPKR and other biomarker CSF levels [26].

In conclusion, we report that pPKR is a biological indicator of the cognitive decline in AD patients that 1- could be utilized to complement the current CSF biomarkers, 2- could be used in future clinical trials, 3 - could represent a possible pharmacological target for future therapeutic approaches [27].
